# Molecular Dynamics Simulation Study of Solid Vibration Permeation in Microporous Amorphous Silica Network Voids

**DOI:** 10.3390/membranes9100132

**Published:** 2019-10-12

**Authors:** Tomohisa Yoshioka, Akihiro Nakata, Kuo-Lun Tung, Masakoto Kanezashi, Toshinori Tsuru

**Affiliations:** 1 Research Center for Membrane and Film Technology, Graduate School of Science, Technology, and Innovation, Kobe University, 1-1 Rokkodai, Nada, Kobe 657-8501, Japan; 2 Department of Chemical Engineering, Hiroshima University, 1-4-1 Kagami-yama, Higashi-Hiroshima 739-8527, Japan; akihiro-nakata@hiroshima-u.ac.jp (A.N.); kanezashi@hiroshima-u.ac.jp (M.K.); tsuru@hiroshima-u.ac.jp (T.T.); 3 Advanced Research Center for Green Materials Science and Technology and Department of Chemical Engineering, National Taiwan University, Taipei 106, Taiwan; kltung@ntu.edu.tw

**Keywords:** molecular dynamics, amorphous silica, gas permeability, gas translation, solid vibration

## Abstract

Microporous silica membranes have silica polymer network voids smaller than 3 Å where only small gas molecules such as helium (2.6 Å) and hydrogen (2.89 Å) can be transported. These silica membranes are highly expected to be available for H_2_ separation. In order to examine gas permeation mechanisms in the silica polymer network voids, factors such as membrane porous structures, gas diffusivity, and gas permeability were studied via membrane permeation molecular dynamics simulation. The thermal motions of silica membrane constituent atoms were examined according to classic harmonic oscillation potential using a suitable amorphous silica structure and non-equilibrium molecular dynamics (NEMD) simulations of gas permeation. The dynamic model successfully simulated the gas permeation characteristics in an amorphous silica membrane with a suitable Hooke’s potential parameter. The introduction of the oscillative thermal motion of the membrane atoms enhanced gas diffusivity. Helium and hydrogen diffusivity and permeability were analyzed using gas translation (GT) and solid vibration (SV) models. The diffusion distance of gas molecules between adsorption sites was around 5.5–7 Å. The solid-type vibration frequencies of gas molecules in the site were on the order of 10^13^ and were reasonably smaller for heavier helium than for hydrogen. Both the GT and SV models could explain the temperature dependency of helium and hydrogen gas diffusivities, but the SV model provided a more realistic geometrical representation of the silica membrane. The SV model also successfully explained gas permeability in an actual silica membrane as well as the virtual amorphous silica membrane.

## 1. Introduction

Microporous amorphous silica membranes have small network voids that are effective for the permeation of small molecules such as helium (kinetic diameter, K.D. = 0.26 nm), neon (K.D. = 0.275 nm), and hydrogen (K.D. = 0.289 nm), which has led to their anticipated application to high-temperature hydrogen separation processes [1]. The separation mechanism of silica network pores is assumed to be so-called molecular sieving, which is based on differences in molecular sizes. Silica network tuning technology has been developed to improve hydrogen permeability [2,3]. Knudsen diffusion, viscous flow [4], and their combined mechanisms [5] have been well investigated for relatively larger pores from several tens of nanometers to microfiltration (MF) regions. Gas transport mechanisms for subnano-scale pores have been theoretically studied, which has involved pores that range in sizes greater than 0.4 nm where smaller molecules such hydrogen can be separated from larger molecules such as methane, light hydrocarbons, SF_6_, or organic hydrides [6,7,8,9]. The authors proposed a modified gas translation (GT) mechanism that explains the properties of gas transport through subnano-scale micropores. This mechanism was useful in characterizing the microporous structures of amorphous silica and zeolite membranes [10,11,12]. Gas transport behavior, an abnormal temperature dependency, and the so-called reversed thermo-switchable molecular sieving phenomenon was observed in molecular sieving membranes composed of flexible metal organic frame (MOF) nanosheets [13]. Organosilica membranes with a high content of organic substituents, such as pyrimidine-bridged organoalkoxysilane membranes, showed molecular sieving performance with activated diffusion of gases, and an affinity to carbon dioxide molecules resulted in a high level of CO2/N2 separation performance [14]. Organic function-based structures also played an important role in deciding the gas permeation characteristics of those membranes.

However, the gas permeation mechanisms through amorphous silica network voids with sizes ranging from 0.2 to 0.4 nm are still not well understood. Knowledge of the effect that network structures and dynamics exert on small gas permeability would be indispensable in the design of molecular sieving amorphous membranes. The authors previously used molecular dynamics (MD) simulations to report that microscopic membrane characteristics, such as void structures and the dynamics of amorphous silica membranes, can have an influence on gas permeation properties for amorphous silica [15,16] and organosilica [17,18] membranes. However, the adequacy of applying the silica network dynamics model to the reproduction and quantitative estimation of actual gas permeation data was not examined in detail. The MD method, however, has not clarified the reliability of the prepared virtual silica membrane models. Hacarlioglu et al. used a quantum chemical calculation of the interaction between silicon and oxygen atoms to estimate the geometrical shapes and sizes of four to eight oxygen-membered siloxane rings that had formed in silica network structures. They reported that the size of an oxygen-membered ring determined the activation energy for gas permeation through silica network voids, and that silica glass had mainly five to six oxygen-membered rings, while six to seven oxygen-membered rings were dominant in silica membranes [19]. These types of quantum chemical calculations involved a relatively small system composed of several tens of atoms, whereas molecular simulation provides data on a molecular scale that are difficult to obtain from actual experiments. 

Molecular transport mechanisms through silica network voids have been used to describe statistical mechanics [20]. A statistical thermodynamic model was derived in the 1970s from gas diffusion and adsorption data in silica glass, and it explained gas molecular transport phenomena as trapping and jumps in small voids (solubility site) formed by a silica polymer network [21]. Oyama et al. showed the validity of the model through mathematical structural analysis and by adjusting the parameters by fitting the model equation to measured gas permeation data. However, parameters in the model, such as effective void space and vibration frequency of permeating molecules, were difficult to measure directly in order to confirm the validity of the estimated values. This statistical dynamics model has not been definitively compared with the empirical activated diffusion/permeation models for subnano-scale microporous materials [7,22]. Thus, the knowledge of gas permeation theory through silica network voids remains insufficient. In order to better establish a gas permeation model for designing high-performance microporous gas-separation membranes, a substantial level of knowledge concerning microporous structures and the kinetic behaviors of gas molecules in small voids seems indispensable. In the present work, gas diffusion and permeation mechanisms through amorphous silica network voids, particularly for gas translation (GT) and solid vibration (SV) models, were clarified quantitatively via non-equilibrium molecular dynamic simulation technique.

## 2. Simulation Method

### 2.1. Modeling of Thermally Vibrated Amorphous SiO_2_ Membranes

**Figure 1:**
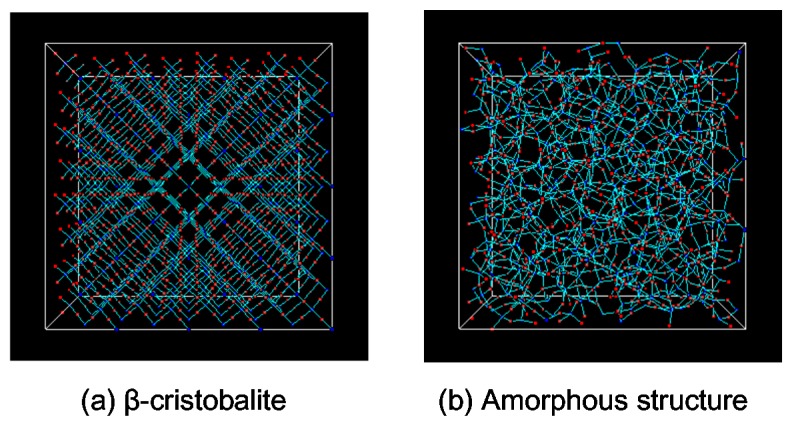
Examples of snapshots of silica structures with a density of 2.2 g/mL; (**a**) β-cristobalite, (**b**) amorphous silica structure.

We have used homemade FORTRAN source program for the present work. Intel^®^ Parallel Studio for Linux was employed as a compiler and the software was run on high performance computers with Intel^®^ Xeon core. The membrane material was amorphous silica that was composed of silicon and oxygen atoms. In order to replicate atomic distances and covalent bond angles, a combination of the modified Born–Mayer–Huggins pair potential and the Stillinger–Weber three-body interactions (BMH–SW) was employed. Details of the force-field functions and parameters can be found in the literature [23,24]. The melt-quench procedure was used to obtain amorphous silica structures with densities of 2.0, 2.1, and 2.2 g/mL. The unit cell size was around (2.9 × 2.9 × 2.9 nm^3^). Figure 1 shows snapshots of silica structures. The density was 2.2 g/mL. The β-cristobalite structure in Figure 1a was melted at 8000 K and quickly quenched to obtain the amorphous silica structure shown in Figure 1b.

Two different virtual silica models were prepared. One was a static model where all atoms were fixed to satisfy atomic pair distances and covalent bond angels of an equilibrated amorphous silica structure. Another was a dynamic model, where the thermal motion or kinetics of atoms was accounted for in addition to the static structure of amorphous silica. 

The inorganic polymer compound in amorphous silica membranes is chemically stable. Those rigid silica membranes are generally assumed not to show dynamic behaviors such as micro-Brownian motion due to the thermal motion of polymer chains. Therefore, the molecular motion that should be simulated in this work is atomic thermal motion that originates from the atomic vibration that is addressed in solid-state physics. Since amorphous silica structures have a short-range order and a chemically stable regular tetrahedral structure, the thermal motion of the atoms on a membrane must be calculated in consideration of the equilibrated location of the atoms in a stable regular tetrahedral structure as base point positions. The thermal motion of atoms in a solid can be approximately described as a simple harmonic oscillation by employing a potential model that contains the natural frequency of an atom as a parameter. In this work, the simplest harmonic oscillation potential model was used. The potential equation is shown as Equation (1).

(1) $$\varphi_{harmonic}=k{\left(r-r^{\prime }\right)}^{2}$$

In Equation (1), *k* is the force constant, *r* is the inter-atomic distance of atom pairs such as Si–O and O–O in the case of silica, *r*’ is the equilibrated distance between the two atoms. This inter-atomic potential model enables us to control the thermal motion that originates from the displacement of an equilibrated position by changing the value of parameter *k* and also allows examination of the effect of the displacement on gas permeation properties. In order to prevent a change in the equilibrated silica network structures by the thermal motion, only the thermal motion of oxygen atoms was considered, and the silicon atoms were fixed at equilibrated positions. The silicon atoms were surrounded by a more electronegative electron cloud of oxygen atoms in an amorphous silica structure, and the interaction between permeation molecules and silicon atoms was assumed to be reasonably negligible [25]. Therefore, fixing silicon atoms at their equilibrated positions on gas permeation properties would amount to a small effect. 

The equilibrated distances between silicon and oxygen atoms, *r*’_Si–O_, and oxygen and oxygen atoms, *r*’_O–O_, were set to be the location of the first peak of the pair of radial distribution functions of the amorphous silica structure. The values of *r*’_Si–O_ = 0.164 nm and *r*’_O–O_ = 0.267 nm were employed. In order to simulate strict atomic motions using the oscillation vibration potential, in addition to the spreading motion of covalently bonded atoms (Si–O), several modes of motion caused by actual thermal molecular motion, such as the angle-changing motion of three-body atoms (O–Si–O, Si–O–Si) and torsion motion that originated from the rotation of atoms, should also be taken into account. However, so many numbers and complexed equations of motion increases computational cost, which makes it difficult to conduct sufficient calculations in order to obtain reliable permeation data for analysis. In the present study, to ease the handling of the thermal motion of the membrane constituent atoms, the vibration of the non-covalently bonded O–O atomic distance was considered to be an alternative motion for the O–Si–O angle movement. Since covalently bonded Si–O atom pairs are more stable than non-bonded O–O atom pairs, more force would be required to restore Si–O harmonic oscillation at an equilibrated location. The detailed differences between these two restoring forces remain unknown, and their optimum values are unclear. Therefore, the spring constant, *k*_O-O_, for O–O atom distance was assumed to be 1/10 of *k*_Si–O_ for Si–O in this work. 

The initial oscillated displacement from the equilibrated position of atom Δ*r* was given according to Equation (2) so that the harmonic oscillation potential satisfied the Maxwell–Boltzmann energy distribution shown in Equation (2).

(2) $$f(\Delta r)=\exp\left(\frac{k{\left(\Delta r\right)}^{2}}{2k_{B}T}\right)$$

In Equation (2), *k*_B_ is the Boltzmann constant, *T* is the absolute temperature, and *k* is the spring constant. The three-dimensional harmonic oscillation is composed of three independent oscillation motions of an atom in the *x*-, *y*-, and *z*-directions. Therefore, the harmonic oscillation potential was calculated for each direction of *x*, *y*, and *z* in this work. The potential parameter, *k*, in Equation (2) is defined as the strength of the chemical bond of atoms. In the case of atomic oscillation due to covalent bonding between the two atoms, the value of k depends on the atomic weight, *m* (kg), and the oscillation frequency, *υ* (s^−1^). The frequency is given by Equation (3).

(3) $$\upsilon =\frac{1}{2\pi }\sqrt{\frac{k}{m}}$$

The frequency, *ν* (s^−1^), is described by the product of the wavenumber, *ω* (1/m), and the propagation speed (speed of light), *c* (m/s). The wavenumber is a unique value that depends on the material that is used, and it can be obtained by spectroscopic measurement. The wavenumber for the covalent bond of Si–O in silica glass has been measured and reported to be *ω* = 700–1100 cm^−1^ [26,27]. In this case, Equation (3) gives *k* = 300–700 J m^−2^. These values of *k*, however, reflect only the stretching oscillation of Si–O, and it might not always be useful for simulating the overall motion of atoms. We employed different values for *k*, such as *k*_Si-O_ = 100, 200, and 300 (*k*_O–O_ = *k*_Si–O_/10), in the same range and order as the above values for trial simulations and empirically decided upon a suitable value of *k* for simulating the performance of actual silica membranes. The thermal motion of an oxygen atom was characterized by calculating the mean squared displacement (MSD) for validation of the harmonic oscillation potential parameter.

### 2.2. Non-Equilibrium Gas Permetion Molecular Dynamics Simulation

Gas permeation simulations were carried out using the dual control plane non-equilibrium molecular dynamics (DCP-NEMD) method [24] under a constant pressure difference and at a constant temperature. Two virtual boundary planes (control plane, CP) of the gas phase were settled at positions in the upstream and downstream sides sufficiently distant from a membrane surface, where permeating molecules were either generated or deleted to control the pressure difference across the membrane. Virtual helium- and hydrogen-like LJ particles, with size parameters of 0.26 and 0.289 nm, respectively, were employed as permeating molecules. For interactions among, and between, permeating molecules and oxygen atoms on the membrane, the well-known Lennard-Jones potential (Equation (4)) was used [7,28,29]. Lennard-Jones potential parameters are summarized in Table 1, where *k*_B_ is the Boltzmann constant. Interaction between an oxygen atom of silica and a permeating molecule was calculated using Lorentz-Berthelot mixing rules given by Equations (5) and (6).

(4) $$\varphi \left(r_{ij}\right)=4\varepsilon_{ij}\left[{\left(\frac{\sigma_{ij}}{r_{ij}}\right)}^{12}-{\left(\frac{\sigma_{ij}}{r_{ij}}\right)}^{6}\right]$$

(5) $$\varepsilon_{ij}=\sqrt{\varepsilon_{ii}\times \varepsilon_{jj}}$$

(6) $$\sigma_{ij}=\frac{\sigma_{ii}+\sigma_{jj}}{2}$$

In the gas permeation simulations of static models, the temperature of the system was controlled via the velocity scaling of guest gas molecules, and the membrane constituent atoms were fixed at equilibrated positions. On the other hand, in the case of dynamic models, the temperature was controlled via the velocity scaling of membrane constituent atoms and no artificial change was added to the velocity of the guest molecules. The time step of MD calculation for the static model was 1 fs, and 0.5 fs was used for the dynamic model. The fifth-order Gear algorithm was used to solve the Newtonian equation of motion for sequential computation of the location and velocity of each atom. The upstream and downstream side pressures were 1 MPa and 0, respectively. The gas permeability, *P* [mol m m^−2^ s^−1^ Pa^−1^], was calculated by counting the number of permeated, and then removed, molecules at the position of a control plane during a steady permeation state [24]. Temperatures of 400–800 K were set to investigate the temperature dependency of gas permeability.

## 3. Results and Discussion

### 3.1. Verification of Silica Models

#### 3.1.1. Harmonic Oscillation Properties

**Figure 2:**
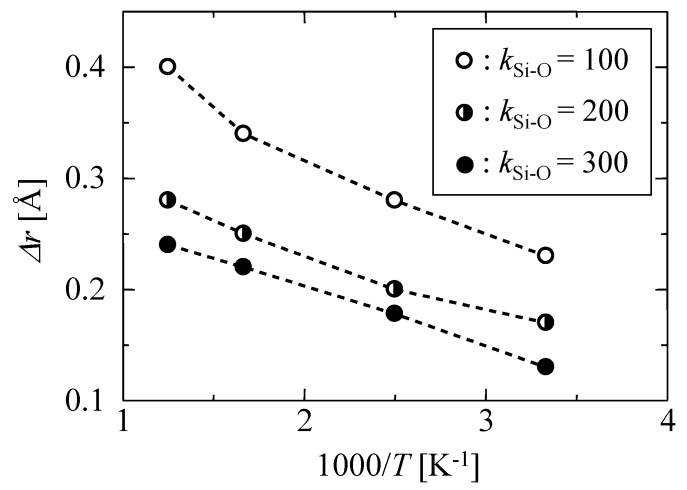
Temperature dependency of the mean displacement of oxygen atoms at different values of *k* parameter.

Figure 2 shows the temperature dependency of the mean displacement of oxygen atoms from an equilibrated position, Δ*r*, as observed for different *k*_Si–O_ values. Under each temperature, the displacement values increased with a decrease in *k*, which indicated that the oscillation displacement caused by the thermal motion could be controlled by changing the value of the *k* parameter. The values of Δ*r* observed in this work and the literature values of several materials at 300 K are summarized in Table 2. The values found in the literature were calculated by molecular simulations using force-field parameters based either on quantum chemical calculation or on the first-principles molecular dynamics method. Therefore, the results were more reliable than our classic MD methods. The values of Δ*r* in this work for *k* = 100–300 J m^−2^ were smaller than that of polyimide [24] but similar to those of silica glass [25,26]. The thermal motion given by a simple harmonic oscillation potential in Equation (1) with *k* = 100–300 J m^−2^ seems to be reasonable for a silica membrane that is an inorganic solid material. 

#### 3.1.2. Effect of the Thermal Motion of Membrane Oxygen Atoms on Gas Permeation Properties

**Figure 3:**
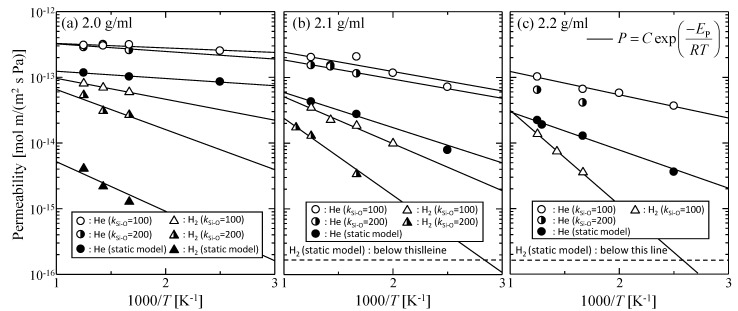
Temperature dependency of gas permeability for virtual silica membrane models of (**a**) 2.0 g/mL, (**b**) 2.1 g/mL, and (**c**) 2.2 g/mL.

Figure 3a–c shows the temperature dependency of gas permeability observed at different virtual membrane densities of 2.0, 2.1, 2.2 g/mL for both the static and dynamic models (*k*_Si–O_ = 100, 200). The permeability ratio of helium and hydrogen (*P*_He_/*P*_H2_) for each condition is summarized in Table 3. The activation energy of permeation, *E*_P_, was also calculated by fitting the temperature dependency of permeability to the Arrhenius equation defined by Equation (7), as shown in Table 4.

(7) $$P=C\exp\left(-\frac{E_{P}}{RT}\right)$$

By considering the thermal motion of membrane constituent oxygen atoms, it was apparent that the gas permeability increased and the activation energy of permeation decreased for all silica membrane density models, which suggested that the thermal motion of the atoms in silica membranes was a structural factor that enhanced gas permeability. The enhancement effect was more conspicuous for a smaller *k* with a larger displacement, Δ*r*, as *P*_He_/*P*_H2_ became smaller. This trend was more obvious for a higher density silica membrane model with fewer permeable paths for larger hydrogen molecules than for those of helium. Hydrogen molecules would be able to permeate smaller voids where only helium could normally be transported due to the enlargement of such voids caused by the thermal motion. As a result, the permeability ratio, *P*_He_/*P*_H2_, possibly became smaller. On the contrary, helium permeability was almost independent of *k* values. For the values of *k* = 100 or 200, the effective path for helium permeation was not remarkably changed. The effect of the thermal motion of membrane atoms on gas permeability depended on both the molecular size of the gas and on membrane porous structures. 

#### 3.1.3. Validation of the Silica Membrane Model

**Figure 4:**
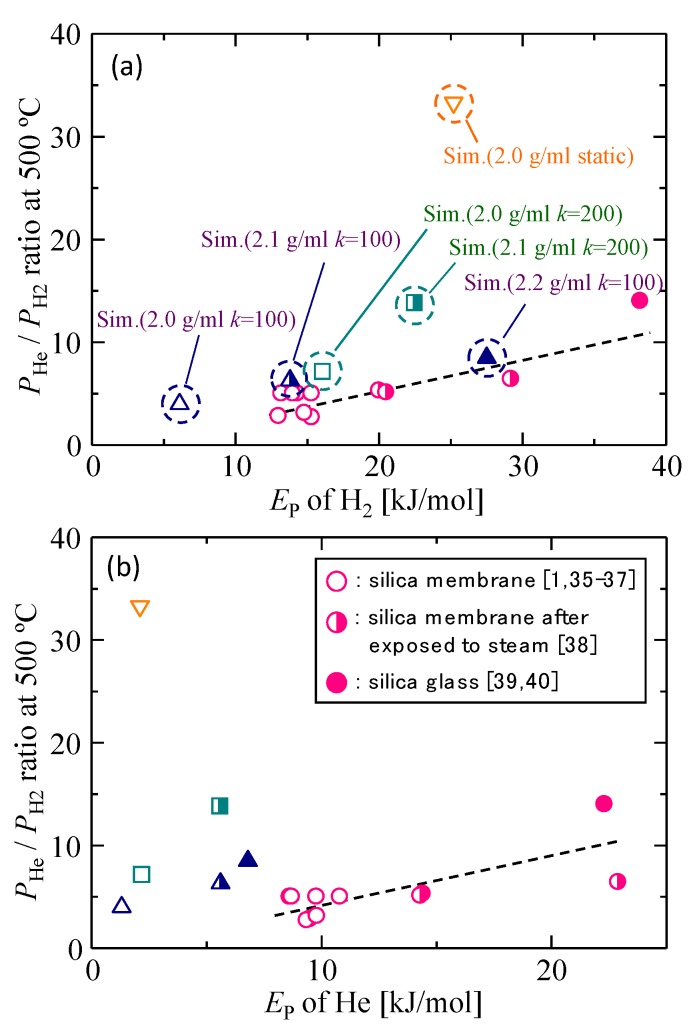
*P*_He_/*P*_H2_ permeability ratio as a function of the activation energies of hydrogen (**a**) and helium (**b**) [1,35,36,37,38,39,40].

**Figure 5:**
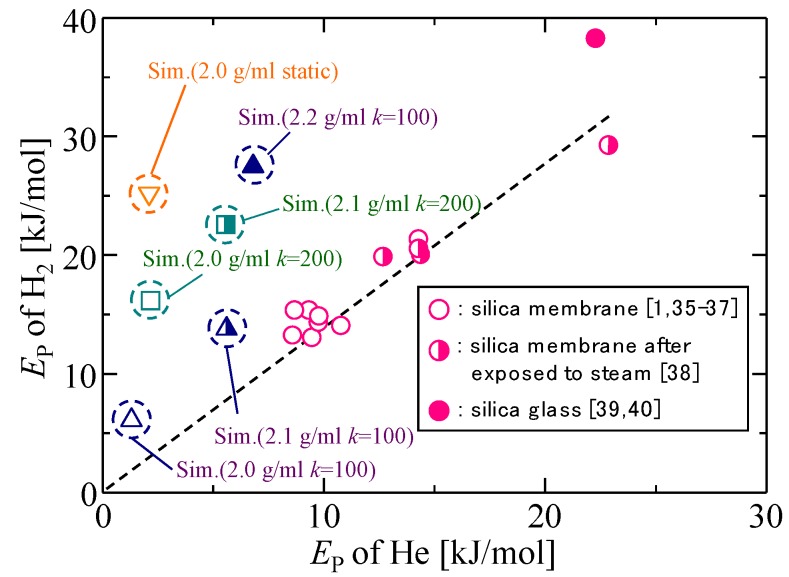
Correlation between the activation energy of helium and that of hydrogen.

The simulated gas permeation properties for different silica membrane models were compared with the gas permeability data of actual silica membranes in Figure 4a,b and Figure 5. Figure 4a,b shows the *P*_He_/*P*_H2_ permeability ratio as a function of the activation energy of each gas molecule, respectively, and Figure 5 shows the correlation between the activation energy of helium and that of hydrogen. Since the *P*_He_/*P*_H2_ permeability ratio and *E*_P_ have higher values in the case of denser membranes with smaller pores, these correlations reflect microporous structures that are detected by permeating gas molecules. Actual amorphous silica membranes can have a variety of gas permeation performances due to the differences in microporous structures, such as the shape of the pore size distribution and the mean pore size, which originates from the difference in the membrane preparation procedure. In each figure, however, good linear correlations were observed for actual silica membranes, which suggested that the properties of actual silica membranes could possibly be plotted according to the linear correlations. The simulation results of dynamic models closely approximated the linear correlations, and the agreement became better for lower values of *k*. The best agreement was observed in the case of the dynamic model with 2.1 g/mL and *k* = 100.

**Figure 6:**
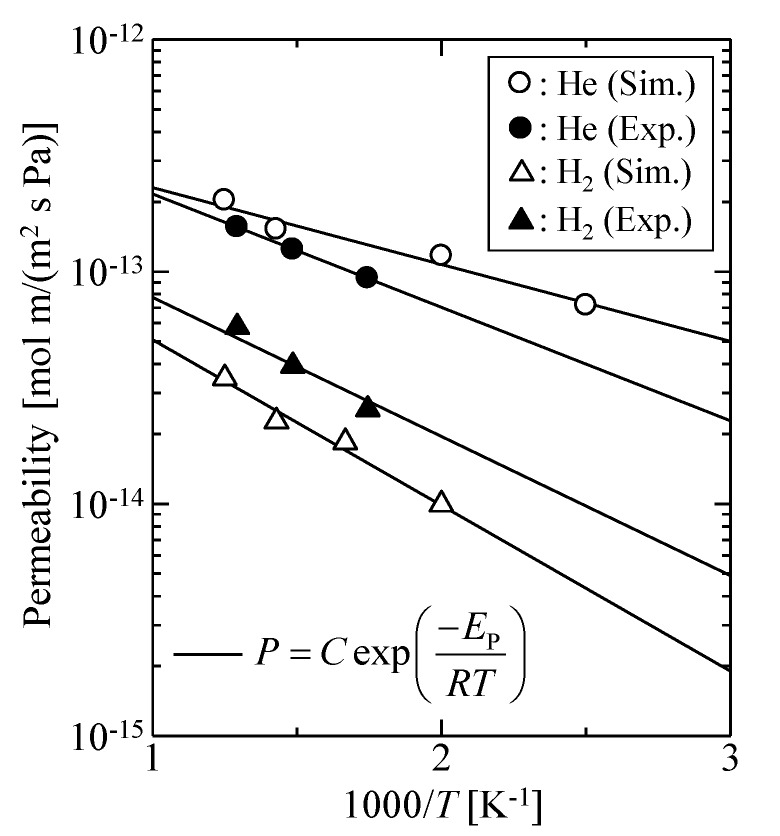
Temperature dependency of gas permeability: Comparison of simulation results and experimental data.

Figure 6 shows the simulated temperature dependency of helium and hydrogen permeabilities for the dynamic model with 2.1 g/mL and *k* = 100 by comparison with the experimental results of an actual silica membrane, where the thickness of the actual silica membrane was assumed to be 150 nm [1]. The simulated gas permeation properties were in good agreement with the experimental data. In this context, the virtual silica membrane model would be reasonable, and we concluded that it could be useful for analysis of the gas permeation mechanisms for an amorphous silica membrane. 

### 3.2. Gas Diffusion and Permeation Models

The permeation flux, *J*, of non-adsorbable gases through a microporous membrane can be described by the well-known Equation (8) [5], where *D* is the gas diffusion coefficient, *ε* is the porosity, and *τ* is the tortuosity.

(8) $$J=\frac{D}{RT}\frac{\Delta p}{L}\frac{\varepsilon }{\tau }$$

Xiao and Wei assumed that the diffusivity of gases in a micropore depended on the ratio of the gas molecular and pore sizes, as well as on the pore shape and the adsorbability of the permeating molecules, *s*, and two different gas diffusion modes were proposed. One was Knudsen diffusion and the other was configurational diffusion (CF). CF describes an activation diffusion for molecular sieving in a molecular-scale micropore [22]. The CF model can well explain the diffusivity of gases in small voids composed of sorption sites and narrow necks with energy barriers that act as permeation resistance in microporous materialism, and the model includes two mechanisms of gas translation (GT) and solid vibration (SV) [22]. Both mechanisms are based on the activated diffusion against a repulsive barrier in a micropore, which can be regarded as a kind of jump between adsorption sites. However, the assumption of molecular kinetics is different for these two mechanisms. The GT mechanism supposes that the molecular kinetics of the jump in a micropore can be approximately modeled as the diffusion of molecules in a gas phase. This GT model can be applicable when the effect of micropore potential on gas transport in a pore is rather small and a molecule is transported based on the molecular velocity in the bulk phase. The diffusivity of the GT mechanism, D_GT_, is given by Equation (9).

(9) $$D_{\mathrm{GT}}=\rho_{g}\alpha \sqrt{\frac{8RT}{\pi M}}\exp\left(\frac{-E_{\mathrm{GT}}}{RT}\right)$$

In Equation (9), *ρ*_g_ is the geometrical factor, the diffusion distance is the distance between adsorption sites, and *E*_GT_ is the activation energy of GT diffusion. 

On the other hand, in the SV mechanism, the motion of molecules constrained by the strong potential field due to a pore wall is assumed to be a kind of solid vibration, in accordance with Hooke’s law. The SV model predicts a restricted movement of gases by the micropore potential in a sorption site with a solid type of vibration. The diffusivity of the SV mechanism, *D*_SV_, is given by Equation (10).

(10) $$D_{\mathrm{SV}}=\rho_{g}\alpha^{2}\upsilon \exp\left(\frac{-E_{\mathrm{SV}}}{RT}\right)$$

In Equation (10), *υ* is the vibration frequency of diffusive molecules, *αυ* corresponds to the jumping molecular velocity, and *E*_SV_ is the activation energy of SV diffusion. 

Molecular diffusion in a micropore, such as small voids in an amorphous silica polymer network, whose size is in the same scale of permeation molecular size can be explained by the Arrhenius-type activated diffusion [7,9,22]. In nature, this activated diffusion is attributed to a molecular transport phenomenon as gas molecules that have kinetic energy that is large enough to overcome the energy barrier in a micropore in order to be transported. According to the GT model, the gas diffusivity depends on both the thermal velocity of a gas molecule based on the kinetic theory of gases, as in the Knudsen model and also on the activation energy for diffusion. On the other hand, in the SV model, the molecular transport is different from a simple thermal motion of gases but is explained as the combination of a stochastic jump velocity of a gas molecule and its activation energy for diffusion. Both the GT and ST models express so-called activated permeation characteristics, but the principal of gas transportation is different. By substituting Equations (6) or (7) for Equation (8), GT gas permeability, *P*_GT_, or SV gas permeability, *P*_SV_, can be obtained using Equations (11) and (12), respectively.

(11) $$P_{\mathrm{GT}}=\frac{JL}{\Delta p}=\rho_{g}\alpha \sqrt{\frac{8}{\pi MRT}}\exp\left(\frac{-E_{\mathrm{GT}}}{RT}\right)\frac{\varepsilon }{\tau }$$

(12) $$P_{\mathrm{SV}}=\rho_{g}\frac{\alpha^{2}\upsilon }{RT}\exp\left(\frac{-E_{\mathrm{SV}}}{RT}\right)\frac{\varepsilon }{\tau }$$

### 3.3. Gas Diffusion and Permeation Characteristics for Silica Membranes

#### 3.3.1. Characterization of a Silica Membrane Structure

**Figure 7:**
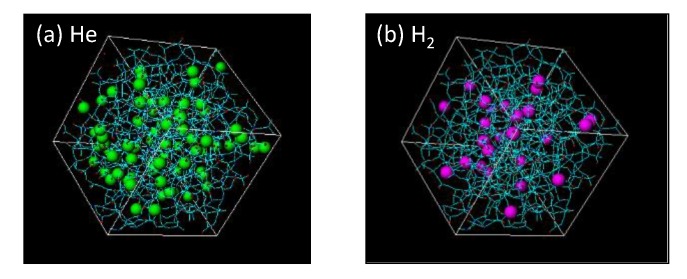
Snapshot of the distribution of sorption sites for probing: (**a**) helium and (**b**) hydrogen.

**Figure 8:**
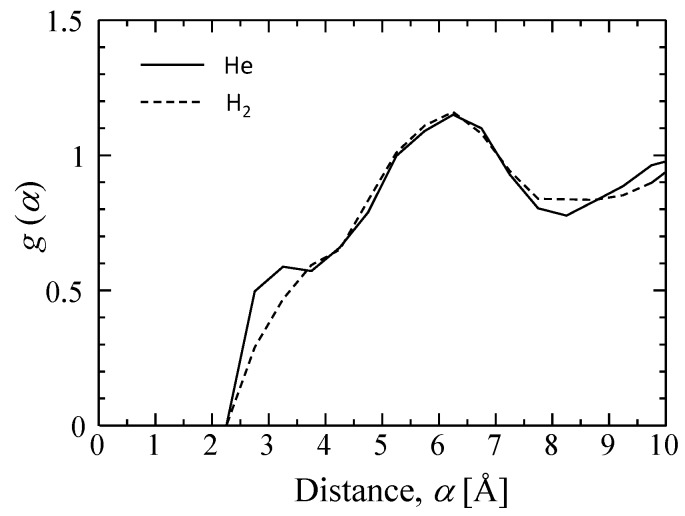
Radial distribution function of sorption sites for probing helium and hydrogen.

Figure 7 features snapshots of the distribution of sorption sites (cage) detected when (a) helium and (b) hydrogen were used as a probing molecule. As shown, the silica structure has more space for the adsorption of smaller helium molecules. Their radial distribution functions (RDFs) appear in Figure 8. At a distance of 5.5–7 Å, peaks larger than 1.0 can be observed in RDFs, and this distance is reasonably assumed to be a jump distance, *α*, from one site to the next for diffusion. The possibility of *α* < 4 Å seems low, but helium would present a higher possibility for diffusion in a shorter distance of approximately 3 Å due to a greater number of site positions for entrapment. 

The detected volumes of each region of the silica membrane model are summarized in Table 5. The overall void volume of a silica polymer network has been defined as the unit cell volume excluding the membrane constituent all-atomic volume. The porosity, *ε*, was estimated based on the accessible volume for each gas molecule. That is, the volume ratio of the sorption site/membrane unit cell, *ε*_He_ = 3.3/19.1 = 0.17 and *ε*_H2_ = 2.5/19.1 = 0.13, respectively.

#### 3.3.2. Gas Diffusivity in a Silica Membrane Model

**Figure 9:**
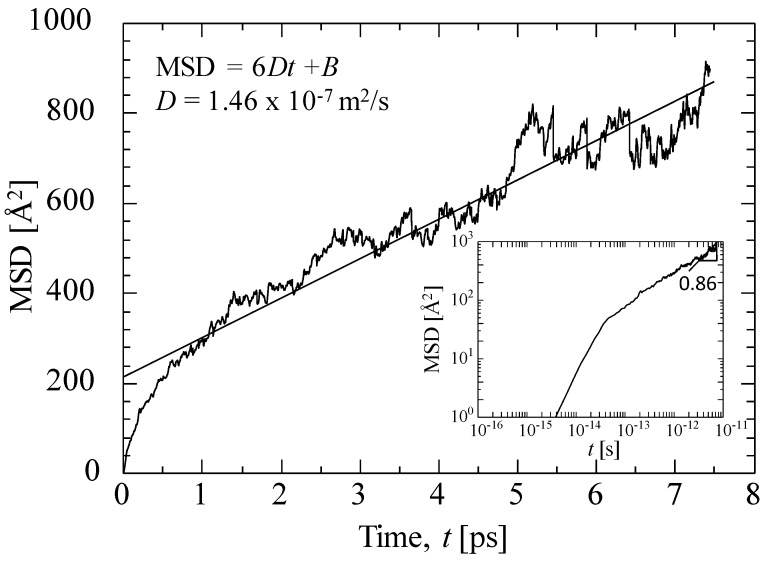
Example of the mean squared displacement (MSD) of helium at 800 K for a virtual silica membrane model (*k* = 100, 2.1 g/mL).

Figure 9 shows the MSD of helium at 800 K for a virtual silica membrane model (dynamic model, *k* = 100) with a density of 2.1 g/mL. The solid line in the figure indicates the fitting of an Einstein equation (Equation (13)) [41] by adjusting the diffusivity, *D*.

(13) $$\mathrm{MSD}=\frac{1}{N}\langle \sum_{i=1}^{N}{\left[r_{i}(t_{0}+t)-r_{i}(t_{0})\right]}^{2}\rangle =B+6Dt$$

In Equation (13), *N* is the number of gas molecules considered in the MSD calculation, *r_i_* (*t*) is the position of gas molecule, *i*, at time, *t*, with *B* as a constant. The diffusivity could be successfully estimated from the slope of the MSD curve. *N* = 50 was adopted in this work in order to obtain a good linear relationship between the MSD and the time. Gas diffusivity in microporous ceramic membranes tends to be greater than that in dense polymeric membranes. The MSD plot on a log scale has also been inserted in Figure 9 and shows the linear increase with time. The slope of the MSD curve in the log–log plot was close to one at *t* > 2 ps. Helium gas molecules were diffused more than 10-fold the distance of their molecular size during an average of seven picoseconds, which can be considered the time range for free diffusion.

**Figure 10:**
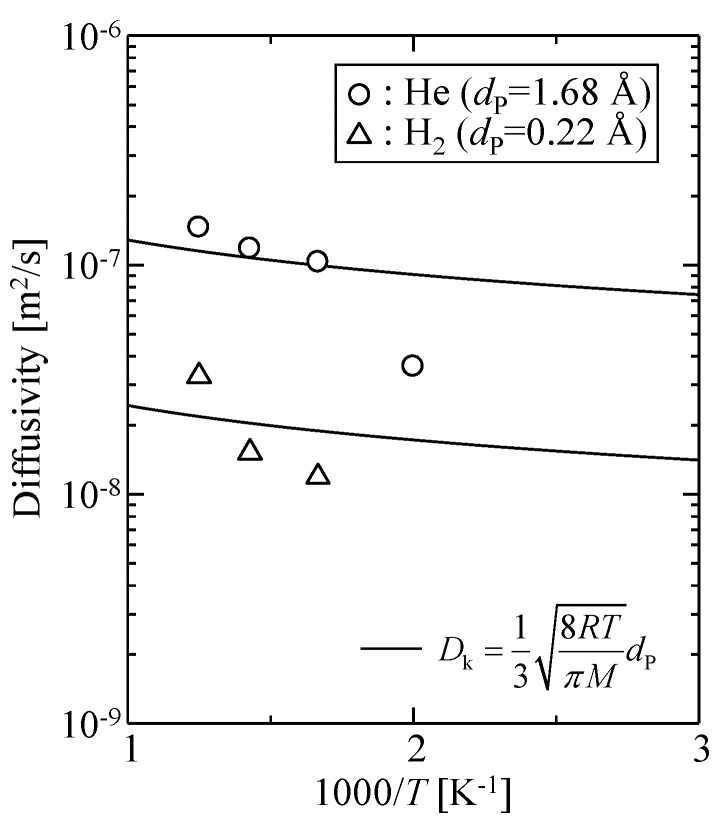
Diffusivities obtained from gas permeation simulations for a virtual amorphous silica membrane model.

Figure 10 shows the calculated temperature dependency of the diffusivity of helium and hydrogen. The solid curves in the figure are fitted Knudsen diffusivity given by Equation (14).

(14) $$D_{K}=\frac{1}{3}\sqrt{\frac{8RT}{\pi M}}d_{P}$$

The adjusting parameter was the pore size, *d*_P_: 1.68 Å for helium and 0.22 Å for hydrogen. The Knudsen model failed to explain the observed diffusivity, and the fitted values of *d*_P_ were too small to serve as an effective pore size for gas permeation. Therefore, the diffusion phenomena of gases in this microporous silica structure were different from gas diffusion in mesopores. 

**Figure 11:**
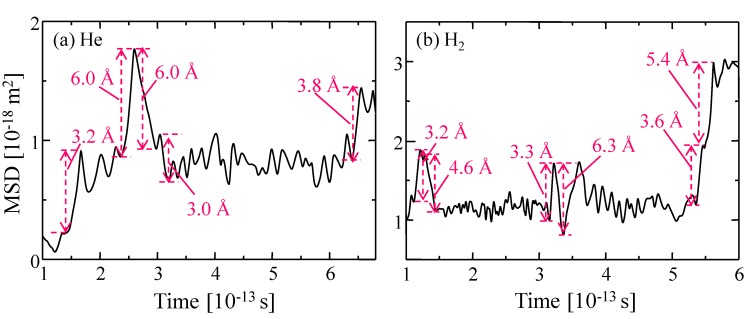
MSD curve of (**a**) one helium and (**b**) one hydrogen molecule obtained from their trajectories as a function of time. The numerical value in the figure indicates a jumping distance directly calculated from their positions at each point in time.

The diffusion behavior of a permeating molecule can be analyzed from the time course of the mean square displacement (MSD), as shown in Figure 11a,b. Two types of vibration of displacement—long-distance displacements greater than 3 Å and shorter ones—can be observed for the MSDs of both helium and hydrogen. The longer ones correspond to jumps in inner-sorption sites and the shorter versions represent intra-cage (sorption site) vibrations of trapped gas molecules.

**Figure 12:**
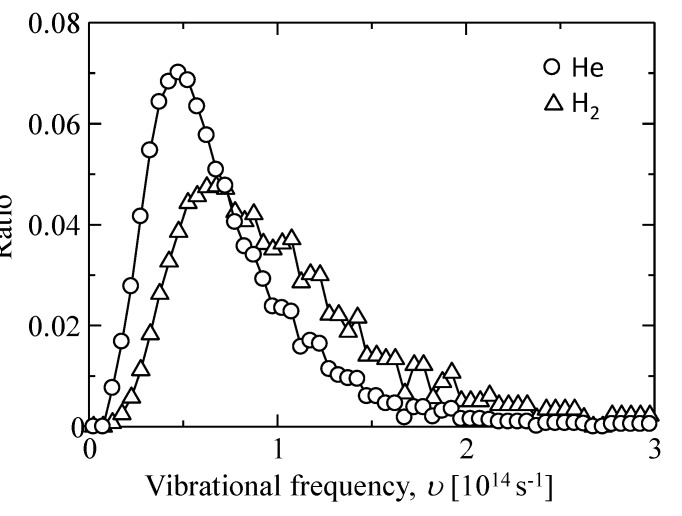
Vibrational frequency distribution of gas molecules trapped in the sorption sites (cage) of an amorphous silica structure.

We assumed that the frequencies of smaller displacements (<3 Å) were guest molecular oscillation frequencies in an adsorption site, which was used to calculate the oscillation frequency, *υ*. The distributions of the values for *υ* of helium and hydrogen are shown in Figure 12. The mode values of frequency were approximately *υ*_He_ = 4.75 × 10^13^ ± 2 × 10^13^ s^−1^ and *υ*_H2_ = 6.75 × 10^13^ ± 2 × 10^13^ s^−1^, which approximated the value of atomic vibration for a solid, 10^13^–10^14^ s^−1^. In addition, *υ*_He_ was higher than *υ*_H2_, and *ν*_H2_/*ν*_He_ was almost (*m*_H2_/*m*_He_)^1/2^ = 1.4. This can be well explained by Equation (3). These results suggested that the diffusing helium and hydrogen in a silica polymer network behaved as if in a solid vibration mode.

### 3.4. Gas Diffusion and Permeation Characteristics

**Figure 13:**
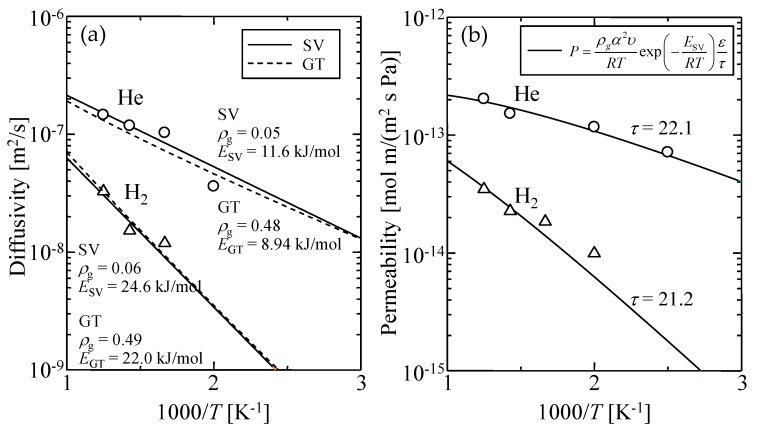
Temperature dependency of simulated (**a**) diffusion coefficient and (**b**) permeability of helium and hydrogen through a virtual amorphous silica membrane.

Figure 13 shows the Arrhenius plots of simulated (a) diffusion coefficients and (b) permeability of helium and hydrogen through a virtual amorphous silica membrane. Both Equations (9) and (10) could be well fitted to the simulated temperature dependence of the diffusion coefficient shown in Figure 13a where the adjusting parameters were the geometrical factor, *ρ*_g_, and the activation energy, *E*_P_. The values of *α* and *υ* were estimated from Figure 8 and Figure 12, respectively, as summarized in Table 6. The values of the adjusted *ρ*_g_ and *E* are shown in Table 7. The possibility of diffusion, *ρ*_g_, is normally defined as 1/3 in three-dimensional bulk space. The value of *ρ*_g_ obtained from Equation (9) was about 0.5 and was too large to represent *ρ*_g_ for the direction of permeation [7], while *ρ*_g_ from Equation (10) was smaller than 0.1. Therefore, the SV model seems more suitable for describing gas diffusion phenomena in silica polymer network voids. 

The solid line in Figure 13b indicates the fitted curve of the SV permeation model given by Equation (12). In this case, since *α*, *υ*, and *ε* can be given, only the tortuosity, *τ*, was the adjusting parameter. The adjusted value of *τ* was about 20, which was in good agreement with the reported value for a microporous inorganic membrane [42], as shown in Table 8. These results suggested that the permeation mechanisms of helium and hydrogen through amorphous silica network voids could be well explained using the solid vibration diffusion model. 

## 4. Conclusions

Harmonic oscillation potential was successfully applied to simulate the thermal motion of atoms that constitute an amorphous silica polymer network. Helium and hydrogen gas permeation simulations through silica membranes were conducted via the DCP-NEMD method, and gas permeation mechanisms and silica membrane structures were analyzed on a molecular scale. The observed temperature dependency of gas permeability for the virtual silica membrane showed good agreement with the experimental gas permeation characteristics in actual silica membranes. The silica membrane model prepared in this work would be suitable for the simulation of actual gas permeation properties. 

The helium and hydrogen diffusivity showed an Arrhenius type of temperature dependency that could not be explained by the Knudsen diffusion model. This indicated that the gas diffusion mechanism in silica polymer network voids is different from Knudsen diffusion that is based on normal gas molecular kinetics. Detailed investigations of an amorphous silica structure and molecular motion in the silica model revealed that the diffusion distance of gas molecules was approximately 6.75 Å depending on the distance between adsorption sites and that the solid-type vibrations of diffusing molecules and their jumps between sites were the dominant factors that determined the gas diffusion mechanisms. The SV gas permeation model sufficiently explained the temperature dependency of gas permeability observed in gas permeation simulations with a reasonably suitable adjusted parameter in the model equation. These results suggested that the SV model provided a better understanding of the gas permeation characteristics of small gas molecules such as helium and hydrogen, which could diffuse and permeate through small voids in the silica polymer network. A virtual silica membrane model of simple harmonic oscillation motion was prepared in this study, which provided molecular dynamics simulations that were useful for the theoretical study of microscopic phenomena on a molecular scale. 

## Figures and Tables

**Table 1:** Lennard-Jones potential parameters.

-	$\varepsilon /k_{B}\;[K]$	[nm]
Helium [7]	10.22	0.26
Hydrogen [28]	38.00	0.289
Oxygen in SiO_2_ [29]	230	0.27

**Table 2:** Oscillative mean displacement of the atoms of several materials at 300 K.

**(a) This Work**	
***k*_Si–O_ [J m^−3^]**	**Δ*r* [Å]**
100	0.23
200	0.17
300	0.15
**(b) Reference**	
**Material**	**Δ*r* [Å]**
Polyimide [30]	1.0
Silica glass [31]	0.28
Silica glass [32]	0.20

The observed oscillation frequency, *υ*, at a steady state, as calculated from the MSD of oxygen atoms, was *υ* = 1.0 × 10^13^ s^−1^, which was in good agreement with the general frequency of solid-material atoms, 10^13^–10^14^ s^−1^ [33,34].

**Table 3:** Permeability ratio, *P*_He_/*P*_H2_, for virtual silica membrane models.

*T* [K]	*P*_He_/*P*_H2_							Knudsen (*M*_H2_/*M*_He_)^0.5^
	Density	2.2 g/mL	2.1 g/mL	2.1 g/mL	2.0 g/mL	2.0 g/mL	2.0 g/mL	
	*k*_Si–O_	100	100	100	200	100	(Static)	
800	-	7.4	11.8	5.8	5.3	3.8	28.8	0.71
700	-	-	-	6.7	10.3	4.3	46.7	
600	-	18.6	33.8	11.8	9.6	5.3	67.0	

**Table 4:** Activation energy of He and H_2_ permeation for virtual silica membrane models.

-		*E*_P_ [kJ mol^−1^]		
		*k*_Si–O_ = 200 [J m^−3^]	*k*_Si–O_ = 100 [J m^−3^]	Static Model
2.2 g/mL	He	-	6.8	11.1
	H_2_	-	27.6	-
2.1 g/mL	He	5.6	5.6	10.2
	H_2_	22.5	13.7	-
2.0 g/mL	He	2.2	1.3	2.1
	H_2_	16.1	6.1	25.2

**Table 5:** Volume portion of the virtual silica membrane structure (2.1 g/mL).

-	Volume [nm^3^]	*ε* [-]
Whole unit cell	19.1	-
Total voids	11.2	0.59
Voids greater than He	3.3	0.17
Voids greater than H_2_	2.5	0.13

**Table 6:** Diffusion distance and vibration frequency of gas molecules in a virtual silica membrane structure (2.1 g/mL).

-	*υ* [10^13^ s^−1^]	*α* [Å]
He	4.75 ± 2.00	6.25 ± 1.00
H_2_	6.75 ± 2.00	

**Table 7:** Values of the adjusted geometrical factor, *ρ*_g_, and activation energy, *E.*

-	-	*ρ*_g_ [-]	*E* [kJ/mol]
GT	He	0.48 ± 0.10	8.94
	H_2_	0.49 ± 0.08	22.0
SV	He	0.05 ± 0.03	11.6
	H_2_	0.06 ± 0.03	24.6

**Table 8:** Values of porosity, *ε*, obtained from the structural properties of the amorphous silica model and the adjusted tortuosity, *τ*, by the solid vibration (SV) model.

-	*ε* [-]	*τ* [-]
He (This work)	0.17	22.1
H_2_ (This work)	0.13	21.2
*Ref.* [30]	0.22	25 ± 5
